# Supplementary material for: Seeking Health in a Digital World: Exploring Immigrant Parents’ Quest for Child Health Information—A Scoping Review
Source: Int J Environ Res Public Health. 2023 Sep 22;20(19):6804. doi: 10.3390/ijerph20196804 (PMC10572919; doi:10.3390/ijerph20196804)
Supplement: Supplementary file 1 [file ijerph-20-06804-s001.zip › ijerph-2561673-supplementary.pdf]

Seeking Health in a Digital World: Exploring Immigrant Parents' Quest for Child Health Information – A Scoping Review

Annina Zysset, Patricia Schwärzler and Julia Dratva

Supplemental Tables

Table S1. Search terms.

Keyword 1	Keyword 2	Keyword 3	Keyword 4
TS=(migrant* OR "documented migrant*" OR immigrant* OR foreigner* OR newcomer*OR "immigrant women" OR "migrant women" OR "immigrant famil*" OR "migrant famil*" OR “migration background”)	TS=(health OR "health info*" OR "health service*" OR "health system" OR "health care" OR "healthcare" OR illness OR disease OR vaccination OR “antenatal care” OR “breast feeding” OR “complementary feeding” OR pseudocroup OR bronchiolitis OR rhinitis OR diarrhea OR infection*)	TS=("digital media" OR "digital technolog*" OR internet OR e$health OR m$health OR "social media" OR facebook OR google OR "information communication technolog*" OR ICT)	TS=(inform OR informing OR use OR using OR utilize OR utilizing OR handle OR handling OR search OR searching OR "information seeking")

Critical appraisals of included studies in the scoping review.

Table S2: Critical appraisal of qualitative studies according to Mays & Pope (2000)

**Articles**	Recto & Champion 2018	Criss et al. 2015	Mason et al., 2020	Qian & Mao, 2021	Gonzalez et al. 2020	Larson et al. 2009
**1 Worth or relevance**						
1.1 Was this piece of work worth doing at all?	2	2	2	2	2	2
1.2 Has it contributed usefully to knowledge?	2	2	2	2	2	1
**2 Clarity of research question**						
2.1 If not at the outset of the study, by the end of the research process, was the research question clear?	2	2	2	2	2	2
**3 Appropriateness of the design of the question**						
3.1 Was an appropriate method used?	2	2	2	2	2	2
**4 Context**						
4.1 Is the context or setting adequately described so that the reader could relate the findings to other settings?	2	2	2	2	2	2
**5 Sampling**						
5.1 Did the sample include the full range of possible causes or settings?	1	2	1	1	1	1
5.2 If appropriate, were efforts made to obtain data that might contradict or modify the analysis extending or modifying the sample?	NC	NC	NC	NC	NC	NC
**6 Data Collection and Analysis**						
6.1 Were the data collection and analysis procedures systematic?	2	2	2	2	2	2
6.2 Was an 'audit trail' provided?	NC	NC	NC	NC	NC	NC
6.3 How well did the analysis succeed in incorporating all the observations?	2	2	2	2	2	1
6.4 Did the analysis develop concepts and categories capable of explaining key processes?	2	2	2	2	2	1

6.5 Was it possible to follow iteration between data and theory?	1	1	1	1	2	1
6.6 Did the researcher search for disconfirming cases?	NC	NC	NC	NC	NC	NC
**7 Reflexivity of the Account**						
7.1 Did the researcher assess the likely impact of the methods used on the data obtained?	1	NC	NC	NC	NC	NC
7.2 Were sufficient data included in the reports to provide sufficient evidence for readers to assess whether analytical criteria were met?	1	2	1	1	2	1

Note: 0 = Low clarity and quality as assesses by the reviewer; 1 = Reasonable clarity and quality as assessed by the reviewer; 2 Reflects a finding of high clarity and quality as assessed by the reviewer; NC = Not clear or not available from the paper. (This does not necessarily mean that it was not addressed by the researchers).

Table S3: Critical appraisal of quantitative studies according to Aromataris et al. (2015)

**Article**	Villadsen et al. 2020	Lee, 2018	Silverman-Lloyd et al. 2020	Sharifi et al. 2020	Reuland et al. 2022
1. Were the criteria for inclusion in the sample clearly defined?	yes	yes	yes	yes	yes
2. Were the study subjects and the setting described in detail?	yes	yes	yes	yes	yes
3. Was the exposure measured in a valid and reliable way?	yes	yes	yes	na	na
4. Were objective, standard criteria used for measurement of the condition?	yes	na	yes	yes	yes
5. Were confounding factors identified?	yes	no	na	na	na

6. Were strategies to deal with confounding factors stated?	yes	no	na	na	na
7. Were the outcomes measured in a valid and reliable way?	yes	yes	yes	yes	yes
8. Was appropriate statistical analysis used?	yes	yes	yes (only descriptive statistics)	yes (only descriptive statistics)	yes (only descriptive statistics)

Note: Na = not applicable

Table S4: Critical appraisal of review studies according to Moola et al. (2000)

**Article**	Hughson et al. 2018
1. Is the review question clearly and explicitly stated?	Yes
2. Were the inclusion criteria appropriate for the review question?	Yes
3. Was the search strategy appropriate?	Yes
4. Were the sources and resources used to search for studies adequate?	Yes
5. Were the criteria for appraising studies appropriate?	na
6. Was critical appraisal conducted by two or more reviewers independently?	na

7. Were there methods to minimize errors in data extraction?	unclear
8. Were the methods used to combine studies appropriate?	Yes
9. Was the likelihood of publication bias assessed?	No
10. Were recommendations for policy and/or practice supported by the reported data?	Yes
11. Were the specific directives for new research appropriate?	Yes

Note. Na = not applicable
